# Fireside Chat on the Asian American, Native Hawaiian, and Pacific Islander Special Edition of the *Health Equity* Journal Featuring Authors

**DOI:** 10.1089/heq.2022.29016.rtd

**Published:** 2022-12-26

**Authors:** Monica McLemore, Zhuo (Adam) Chen, Elle Lett, Raynald Samoa, April Moreno, Bei Wu, Leena Yin, Donglan “Stacy” Zhang, Wanda Montalvo, Celine Nguyen

**Affiliations:** ^1^ Editor-in-Chief, Health Equity, and Interim Director of the University of Washington's Center for Antiracism in Nursing, Seattle, Washington, USA.; ^2^ Associate Professor of Health Policy and Management, College of Public Health, The University of Georgia, Athens, Georgia, USA.; ^3^ Perelman School of Medicine, University of Pennsylvania, Philadelphia, Pennsylvania, USA.; ^4^ Endocrinologist at the City of Hope, Duarte, California, USA. Member, President's Advisory Commission on Asian American, Native Hawaiians and Pacific Islanders; Co-Chair of the Data Disaggregation Subcommittee.; ^5^ Founder of the Public Health Podcast and Media Network and the Autoimmune Community Institute, California, USA.; ^6^ Dean's Professor in Global Health, Vice Dean, Research, Affiliated Professor, Ashman Department of Periodontology & Implant Dentistry, Co-director, NYU Aging Incubator, New York, New York, USA.; ^7^ Swedish Cherry Hill Family Medicine Residency in Seattle, Washington, USA.; ^8^ Associate Professor in the Division of Health Services Research, Department of Foundations of Medicine at the New York University Long Island School of Medicine, Mineola, New York, USA.; ^9^ Senior Fellow and Team Lead Public Health Integration and Innovation, National Association of Community Health Centers (NACHC), Clinical Affairs Division, Bethesda, Marylane, USA.; ^10^ Rice University, Houston, Texas, USA.

## Expert Panel


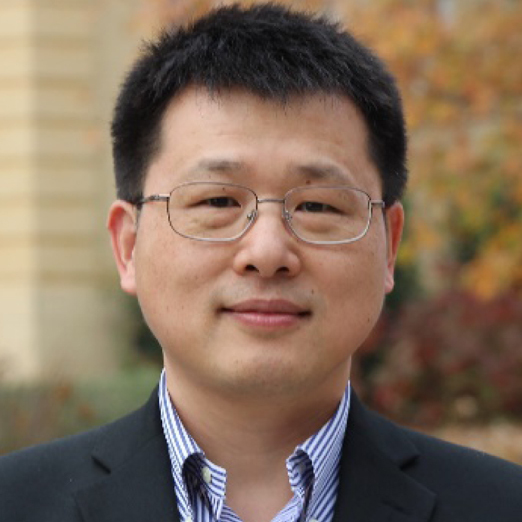


**Dr. Zhuo (Adam) Chen (moderator)** is associate professor and DrPH program coordinator, Department of Health Policy and Management, College of Public Health, University of Georgia (UGA), Athens, Georgia, USA, visiting professor of health economics and director of academics (0.2FTE), Centre for Health Economics, School of Economics, University of Nottingham Ningbo China. Dr. Chen leads the Interdisciplinary Approaches to Social Determinants of Health Pre-Seed Team at UGA and coleads the Health Disparities Research Workgroup within the UGA College of Public Health. He earned his PhD in economics and MS in statistics from Iowa State University. Before Dr. Chen joined the University of Georgia, he was a senior health economist with the U.S. Centers for Disease Control and Prevention (CDC). He has been a guest researcher since 2017 with the CDC Office of Genomics and Precision Public Health. Dr. Chen is affiliated with the Atlanta-based China Research Center and the Core China Research Group—Universidad de Navarra. Dr. Chen's current research interests include health economics, economics of genomics, social determinants of health, global health, health systems, mental health, and economic evaluation. He has published more than 100 peer-reviewed publications. His works have appeared in *Lancet*, *JAMA Network Open*, *Health Policy*, *Health Economics*, *Journal of Health Economics*, *Genetics in Medicine*, and *Social Science & Medicine*. He was a recipient of the CDC Excellence in Social and Behavioral Science Research Award in 2013 for his work on examining the role of geographic scale in testing the income inequality hypothesis. He earned the Excellence in Diversity Award (Civilian) from the Federal Asian and Pacific Americans Council in 2016 for his work promoting diversity and inclusion at CDC. He serves as an associate editor of *Health Equity*, academic editor of *PLoS One*, and on the editorial board of *Journal of Family and Economic Issues*, *China CDC Weekly*, and *Global Health Research and Policy*.

**Twitter:** @Zhuo_Adam_Chen


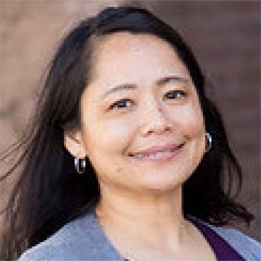


**Dr. April Moreno** is trained in public health, informatics, and public administration and is based in southern California. Dr. Moreno is the founder of the Public Health Podcast and Media Network as well as the Autoimmune Community Institute, an organization dedicated to autoimmune health equity.

**Twitter:** @PHPodcasters


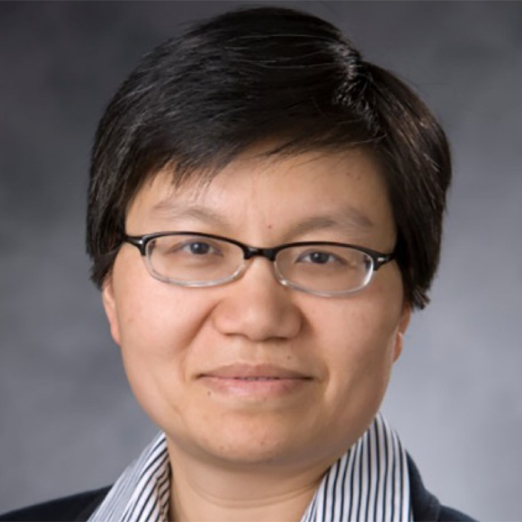


**Dr. Bei Wu** is vice dean for research and dean's professor in global health at the NYU Rory Meyers College of Nursing. She is also cofounder of the NYU Aging Incubator. Dr. Wu is currently leading several NIH-funded projects including a clinical trial to improve oral health for persons with cognitive impairment. She coleads the Rutgers-NYU Center for Asian Health Promotion and Equity. Through this center, she leads a 5-year intervention study that focuses on supporting Chinese and Korean dementia caregivers. Dr. Wu is a principal investigator on the NIA-funded Asian Resource Center for Minority Aging. Her extensive publications cover a wide range of topics including risk factors related to cognitive impairment, dementia caregiving, and geriatric oral health. Dr. Wu is a fellow of the Gerontological Society of America and the New York Academy of Medicine. She is also an honorary fellow of the American Academy of Nursing.

**Twitter:** @beiwu66


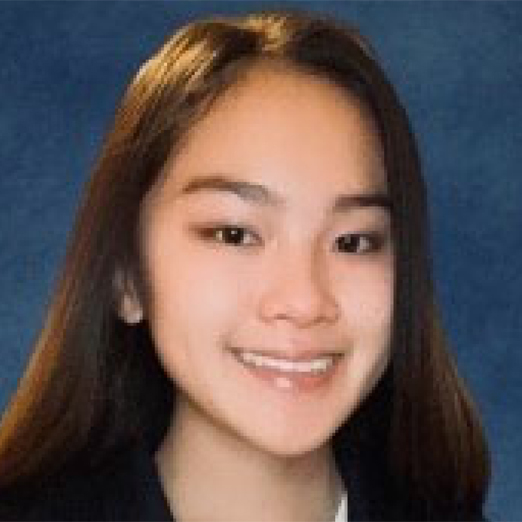


**Celine Nguyen** is a recent graduate from Rice University with a BA in psychology and has worked with underserved populations in research, nonprofit, and volunteer roles. She is dedicated to facilitating cross-disciplinary collaborative opportunities to tackle issues of health, well-being, and social inequities. Currently, Celine serves as a research assistant for an NIH Community Engagement Alliance (CEAL) project at UH with the goal to address COVID-19 health disparities affecting racial and ethnic minority populations.

**Twitter:** @Celine_D_Nguyen

**Dr. Elle Lett (moderator),** is a black, transgender woman, statistician-epidemiologist, and physician-in training. Through her work, she applies the theories and principles of black feminism to understanding the health impacts of systemic racism, transphobia, and other forms of discrimination on oppressed groups in the United States. She holds a PhD in epidemiology from the University of Pennsylvania, master's degrees in statistics and biostatistics from The Wharton School and Duke University, respectively, and a bachelor's degree in molecular and cellular biology from Harvard College. To date, her work has focused on intersectional approaches to transgender health and the health impacts of state-sanctioned violence and other forms of systemic racism. Now, she is turning her focus to algorithmic fairness in clinical prediction models and mitigating systems of inequity in health services provision. She is engaging in this new arm of research through a postdoctoral fellowship at the Boston Children's Hospital Computational Health Informatics Program (CHIP), before returning to finish her clinical training.

**Twitter:** @ElleLettMDPhD


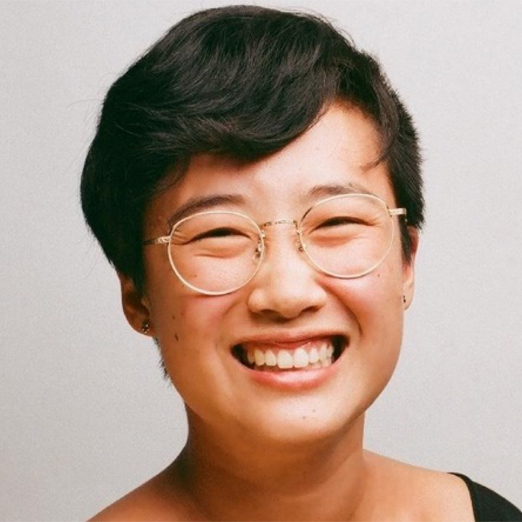


**Dr. Leena Yin** is a PGY-1 at the Swedish Cherry Hill Family Medicine Residency in Seattle, WA, where she cares predominantly for communities of color, particularly in the Seattle Chinatown-International District. She graduated from the University of California, San Francisco, where she served as advocacy vice president for the National Asian Pacific American Student Association.

**Twitter:** @lyyinaround


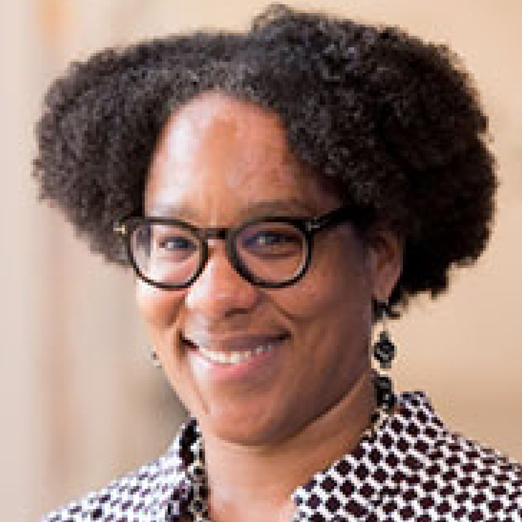


**Dr. Monica McLemore (moderator)** is a tenured professor in the Child, Family, and Population Health Department and interim director for the Center for Anti-Racism in Nursing at the University of Washington School of Nursing. She retired from clinical work in 2019; however, she currently provides flu and COVID-19 vaccines. Her research is focused on reproductive justice. Her peer-reviewed articles, Op-eds, and commentaries have been cited in five amicus briefs to the Supreme Court of the United States and three NASEM reports. She became editor-in-chief of *Health Equity* in 2022.

**Twitter:** @mclemoremr


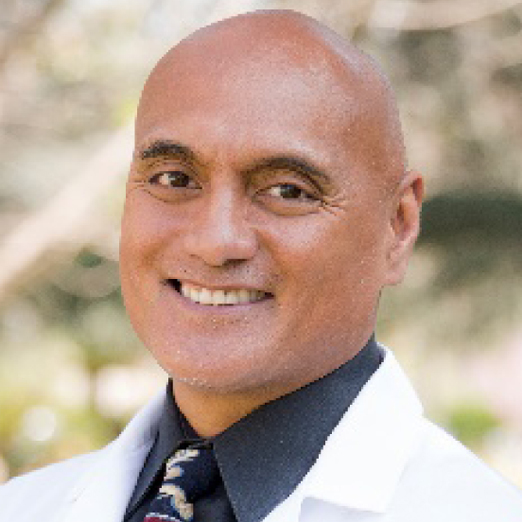


**Dr. Raynald Samoa** is a graduate from the University of Washington School of Medicine. He completed his residency and fellowship training at USC Los Angeles County General Hospital. He is currently an endocrinologist at the City of Hope. Dr. Samoa served as the lead for the National Pacific Islander COVID-19 Response Team and has authored several articles describing the impact of COVID-19 on Pacific Islander communities. He has testified to the House of Representative Ways and Means Committee during a session entitled *THE DISPROPORTIONATE IMPACT OF COVID-19 ON COMMUNITIES OF COLOR.* Dr. Samoa serves on the President's Advisory Commission on Asian American, Native Hawaiians, and Pacific Islanders and is the cochair of the Data Disaggregation Subcommittee. He currently is the technical advisory lead for the Healing Association of Pacific Islander Physicians and the Data and Research Council of the National Association of Pacific Islander Organizations (NAOPO).

**Twitter:** @DrRaySamoa


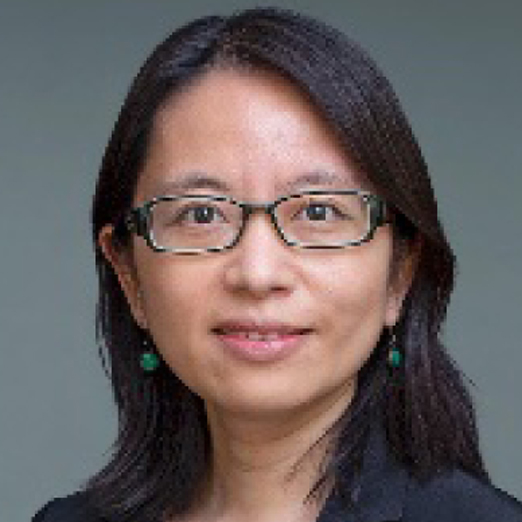


**Dr. Donglan “Stacy” Zhang** is an associate professor in the Division of Health Services Research, Department of Foundations of Medicine at the New York University Long Island School of Medicine, with expertise in population health management, health policy and economics, health systems research, and comparative effectiveness research. Dr. Zhang's research focuses on understanding and addressing health disparities in chronic diseases by investigating barriers to accessing medical care and healthy food, studying multilevel social determinants of health (e.g., health insurance, food policy, racial discrimination) that affect health outcomes, and advancing knowledge in racial minority health and rural patient care. Dr. Zhang is principal investigator and co-investigator of multiple NIH, CDC-funded grants that investigate disparities in cardiovascular disease outcomes, and use survey, claims, and electronic medical records to evaluate the effectiveness of clinical interventions, community-based interventions, and health policies on narrowing the gaps in disease prevention and treatment.

**Twitter:** @SH_LA_AT


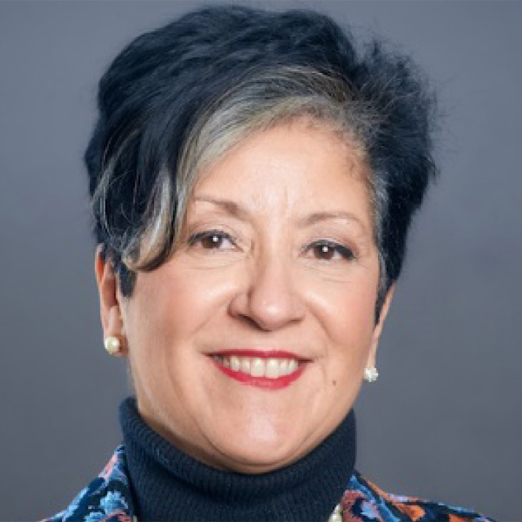


**Dr. Wanda Montalvo** is a senior fellow for Public Health Integration, and Innovation at the National Association of Community Health Centers (NACHC), where she provides technical support for public health systems change with a focus on peer learning, health disparities, health equity, and transforming new models of care. Dr. Montalvo is an innovative nurse leader and avid public health advocate with a successful track record of engaging diverse stakeholders from academia, philanthropy, federal agencies, and community-based organizations.

**Twitter:** @Montalvo501

***Dr. Lett:* My name is Dr. Elle Lett. I am the social media editor for *Health Equity* journal, and I am really excited to be here with you today. We are having this special fireside chat for the Asian American, Native Hawaiian, and Pacific Islander (AANHPI) special edition of *Health Equity*. Dr. Zhuo Adam Chen from the University of Georgia, who was the guest editor for that edition, has helped organize some of the authors to come and talk about their work with a group of individuals online.**

**Dr. McLemore, our editor-in-chief, is also present. I will ask her to give a bit of an introduction and then we can discuss some ground rules for the session.**

***Dr. Mclemore:* Welcome everyone. I am so grateful that you are here for this fireside chat, an informal discussion with some of the authors from a special edition that was published in *Health Equity* both online and print. I will let Dr. Chen talk a little bit about why that was done. I am so grateful that you all are taking some time out this morning and afternoon and evening to spend with us.**

**This is being recorded because we do know that there are many people who set reminders and wanted to participate. We wanted to be able to make sure that it would have the widest range of audience possible. We are very fortunate and lucky, not only to have the guest editor of this special edition, Dr. Zhuo Adam Chen from the University of Georgia with us, but to have many of the coauthors as well.**

**I am super excited to introduce my coeditor. We wrote in an opening editorial to introduce this issue. But instead of speaking for him and the rest of the authors, this was a huge amount of work and I want to thank him publicly. We had a ton of submissions. And for those people who do not know, *Health Equity* can only accommodate 15 articles in any category of articles that we accept for a special edition.**

**So, we were, unfortunately, not able to publish every article in the special edition. But I will say that to date, two of the articles that were submitted are being published in the regular edition of *Health Equity*. So, with that, I am going to turn it over to Dr. Zhuo Chen, who was on board at *Health Equity* before I was brought in as the editor-in-chief and really got me up to speed really quickly because the process was already in place and just he was able to really pull together a stellar group of early career, mid-career, and career scientists to talk about the very important issue of AANHPI health.**

***Dr. Chen:* Thanks, Dr. McLemore for the introduction. And thanks to Dr. Lett for hosting this event. Yes, this especially is really a collective effort including Dr. McLemore, and Dr. Grace Ma, and the former acting Editor-in-Chief Dr. Xinzhi Zhang from NIH. During the height of the pandemic, we have seen that AANHPIs have really been hit hard by the pandemic, and by hate crimes toward the Asian American community.**

**That is the reason we wanted to investigate how this pandemic has impacted the different aspects of life for the health of AANHPIs. I also want to thank the support of the Rockefeller-endowed China Medical Board, an American Philanthropy, and the Blue Shield of California Foundation, which funded this special issue.**

**As Dr. McLemore mentioned, we have both online and print copies of this special issue, which, with funding from Blue Shield of California Foundation, we are going to send to every member of congress and other stakeholders, so that we can illustrate the predicaments of AANHPIs during this pandemic and highlight the research of our authors. I want to thank you all for contributing to this special issue.**

**Dr. Wu:** I am Bei Wu, dean's professor in global health at NYU Rory Meyers College of Nursing. I jointly worked with Dr. Wei Zhang and Yan Yan Wu from University of Hawaii on the racial and ethnic disparities in getting a COVID-19 vaccine. We examine this disparity in COVID-19 vaccination rate by age among white, Hispanic, blacks, and Asian Americans and the effects of gender and education.

Our study used seven waves of biweekly surveys collected between July 2021 and October 2021. Our study found that Asians reported the highest, while blacks report the lowest vaccination rate, but gender differences were minimal.

But, very interestingly, we find that there is a huge heterogeneity within different age groups among Asian populations. While Asians overall had the highest vaccination rate, those individuals aged 80 years and above have one of the lowest vaccination rates.

Also, we want to particularly highlight the significant impact of education on vaccination rates across racial and ethnic groups. But the caveat of this study is there is huge heterogeneity within Asian American populations. With these data, we are not able to tease out the different groups among Asian Americans because we do not have the data available.

The second major issue with this data set is that the surveys were conducted in English, which systematically excludes those individuals with limited English proficiency.

When you look at the census, more than half of Asian Americans, in particular older Asian Americans, have very limited English proficiency. So overall, this kind of data excludes those individuals who potentially have a lower socioeconomic status, who may also have limited access to these surveys, vaccination, and health care services.

***Dr. Mclemore:* Dr. Wu, can you briefly tell folks the data and the survey that you use? Because I think your point about disaggregation is very important. Also, are there national and/or regional efforts similar to the *We are Greater than COVID* project that have specifically targeted information in native languages for individuals about COVID-19, at least vaccination precautions, and/or COVID-19 vaccination information?**

**Dr. Wu:** Thank you for raising this question. The study that we use is called the Household Plus survey. This is an ongoing cross-sectional survey developed by the U.S. Census Bureau and the National Center for Health Statistics to measure how a household experienced COVID-19. This survey was conducted in English. In terms of the second question, so as far as I know that in terms of a national survey, there is no particular effort to target Asian Americans that speak their native languages. New York Department of Health may collect some data on that at the local level.

***Dr. Lett:* Your response has mirrored some of the things that I was going to bring up. Whenever I think about a study, I think about what you observed and then what is the truth. What you observed obviously is already challenging when you have the most vulnerable population within the Asian community being the older population based on the other risk factors, especially with COVID-19.**

**And they are the ones with the least vaccination, so that, to me, highlights a public health intervention failure where we really need to have that population vaccinated. Then, you think about what the truth could be based on the data limitations where you cannot disaggregate. People who have language barriers are also less likely to have had the vaccination.**

**Then, it is possible that what you observe is actually biased from more severe inequities, where you might even have even worse vaccination status among some of those groups, particularly the older groups. And then you might be undercounting people who have language barriers, so that the high vaccination rate among Asian populations might actually be slightly or even more than slightly overestimated.**

**That makes me think about how disaggregating data is actually such a fundamental public health intervention for so many populations. When you think about other racial ethnic groups like the Latinx Hispanic group and even the African diaspora and then when we think about gender and trans people that there is a recurrent theme that we see in *Health Equity* the journal and health equity broadly where the sociopolitical process of categorization, because remember the census is created by a process that is political, often does a disservice to those of us who are in those marginalized groups.**

**Thank you for this work, and it is interesting to see you wrestling with some of the same data challenges for the AANHPI communities that I wrestle with for other communities.**

**Dr. Wu:** Absolutely. That is one of the major struggles that we have when we are interested in this population. Our team has used many national data centers trying to address some of the health outcomes among Asian Americans. And just to give you a bit more detailed information from this study, when you look at those aged 80 years and above, this study only covers 80 to 88 years, when we know as a nation that the oldest are the most vulnerable to COVID-19. For whites in this older population, the vaccination rate is 84%. For Asian Americans, it is 69%. For Hispanics, it is even lower, 41%.

And for blacks, it is 89%. So, you can see this is a huge disparity within these older minority populations in the nation. Even for those individuals who speak English well enough to be included in this survey, you clearly see some disparities there. But certainly, there is some other factors that can potentially contribute to this as well, including access to care.

***Dr. Lett:* But it is also a household study, right? And so that means that if the ascertainment criterion is to be in the household, you are totally leaving out people who experience housing insecurity or who are just outright experiencing homelessness.**

**So, that is another layer of undercounting people within the Asian community who might not be vaccinated.**

**Dr. Wu:** Absolutely. Yes.

**Ms. Nguyen:** My name is Celine Nguyen. I am a recent graduate from Rice University and a representative of Boat People SOS Houston, a nonprofit social health and legal service provider whose purpose is to empower, organize, and equip immigrant communities.

I am also a research assistant and author for one of the NIH CEAL projects led by Dr. Lauren Gilbert and Dr. Bich-May Nguyen at the University of Houston College of Medicine. We investigated the facilitators and barriers to COVID-19 vaccinations and clinical trials in the Vietnamese American community in Houston, Texas, through a qualitative study.

This is a population that is under-researched. And, as it was mentioned earlier, data on specific communities in the Asian American population are often aggregated and can mask community-specific health needs or health behaviors. So, this was one of our motivating factors for this study. We specifically conducted focus groups and key informant interviews between August and October 2021 using a community-engaged approach.

We utilized community organizations to help us recruit Vietnamese American adults in the Houston area who could choose either English or Vietnamese for the survey, focus groups, and interviews. For key informal interviews, we recruited faith leaders, health care workers, and small business owners for broad community perspectives. In our findings, a few overarching themes influencing participants decision making around COVID-19 vaccination and clinical trial participation emerged, including language, generational differences, and a collectivist approach.

In studying those populations' willingness to vaccinate against COVID-19, risk of severe illness, ease of access, trust in science, and having trusted sources of information, specifically in culturally translated Vietnamese, also arose as important facilitators. Participants reported barriers to vaccines such as concerns about vaccine safety, lack of health care access, and misinformation.

This is just summarizing our findings. We are working on finalizing another article focusing on Vietnamese Americans' attitudes and beliefs around COVID-19 and then an article summarizing our outreach campaign. Again, thank you for inviting me to speak on behalf of Dr. Bich-May Nguyen and Dr. Lauren Gilbert.

***Dr. Mclemore:* Can you talk a little bit about the methodology that you use and what drove your methodology? I think that it is really innovative.**

**Ms. Nguyen:** The questions were from part of the NIH CEAL common survey. For our data analysis, we followed a thematic analysis approach, where different individuals in our research team reviewed the notes and listened to the audio recording. And because we wanted to ensure that we had a more representative sample, these groups and interviews were held in both Vietnamese and English in separate sessions.

So, we had to translate the Vietnamese sessions to English to accurately capture the conversations, and independently code at each session using an inductive coding approach. We derived preliminary codes from the data, and then we met with the research team to discuss emerging codes and ongoing themes. Through this iterative group approach, the final set of codes and themes was agreed upon by the research team using the software NVivo.

Then, they were validated and refined after working with feedback from our community partners with BPSOS Houston and Pivot. The resulting themes were representative of the patterns of meetings that we discovered through both the interviews and the focus groups.

***Dr. Mclemore:* Thank you for that. What I really loved about this was that your use of mixed methods. I thought it was a nice exemplar for people who are interested in wanting to use both key informant interviews and group data. You wrote it so beautifully in terms of how you were able to triangulate those data.**

***Dr. Lett:* I want to highlight how perfect a marriage Ms. Nguyen's and Dr. Wu's articles are. Of course, different epistemologies, quantitative and qualitative work, but it is almost like Ms. Nguyen's work is a response to some of the limitations of the surveys that are used in work like Dr. Wu's.**

**I really appreciate having you both go back-to-back to show that the quantitative data is broad strokes under these limitations and then this qualitative data can allow you to dive deeper within the population and get at disaggregation and more nuanced. All I wanted to add is whether there are any particularly salient quotes that you recall that you remember that you wanted to highlight here.**

**Ms. Nguyen:** I think one of the most important things that we found, and I think Dr. Wu mentioned this, was the theme of generational differences. There are some individuals who reported that language barriers impacted older Vietnamese people's access to credible COVID-19 and vaccination information, which I believe was mentioned by Dr. Wu in her research as well.

One other really interesting thing that we found was that many of our younger participants reported cultural influences, such as the significance of familial hierarchy and respecting the elders, affecting productive discussions on COVID-19 misinformation with their families and community.

**Dr. Montalvo:** I work with the National Association of Community Health Centers; COVID-19 keeps me up at night. I am really interested in the Asian American and Pacific Islander (AAPI) communities because I do understand that there has been a lack of information in multiple languages. Public health infrastructure in the various states needs additional support. I am interested in how the researchers have come back to the community with their findings, and how do you do the handoff so that there is some type of activation with the information you are sharing back? What has worked? What are strategies that we need to think about to move the research to next steps?

**Ms. Nguyen:** I think one of the factors that really helped us in outreaching this information to the community is by involving community partners from the beginning of our research. A representative from BPSOS Houston was present on all our research meetings. Afterward through their connections with other local AAPI communities, and specifically, Vietnamese American communities in the greater Houston area, we were able to share our data.

We found that there was a barrier of getting the youth vaccinated in our community, and that prompted a few community organizations to focus their efforts on reaching this community. Using our data from the focus groups and the interviews, we worked with Pivot and BPSOS to create a multimedia outreach campaign using newspaper sources and internet ads to outreach on COVID-19 vaccinations to this community.

***Dr. Lett:* I love that by having them from the beginning, you can actually shorten the time it takes before the findings can be directly implemented.**

**You have made such a beautiful case for community-based participatory research and qualitative methods.**

**Ms. Nguyen:** Thank you for your statement. I just wanted to say that I think by involving BPSOS Houston, they actually helped to guide our research in the first place. So, by incorporating their views and their previous work with the community, we were able to identify a way to get more participants. And I believe that partnership was necessary for the success of our project.

**Dr. Wu:** This is very well said. From a research perspective, this is particularly the case given the fact that there are so few large representative surveys available that can answer some of the really important questions about the AAPI population. We need to work with community organizations to guide our research and come up with some research questions. For example, the community partnership is really a key for us to work in this field that this partnership needs to start from the beginning to think about community input.

There is a codesign of research protocol, codesign for research approach, then help with recruitment and input on questionnaire development. Also, think about how they can help us interpret data as well as actually help disseminate research findings.

But, in addition, to inform policy at the national level, we still need to have a large data set moving forward, we need to think in terms of how we could potentially push for initiatives that start to focus on the major ethnic groups within Asian populations using native languages.

Otherwise, we keep talking about this, but nothing happens. We should think about a multipronged approach at the national level as well as at the local level.

**Dr. Moreno:** I am Dr. April Moreno. I founded the Public Health Podcast and Media Network last year. A lot of it was COVID-19-motivated to start because of the misinformation that was out there. But, we are also part of the AAPI community as well as the Latinx and the black communities. Thank you for hosting this space today and this conversation. I am grateful to hear that AAPI and health equity are being addressed in the research and in your journal. Thank you for talking about the data disaggregation that continues to need to be done.

When we look at things like socioeconomic status, for example, it is a huge issue. Within the Southeast Asian population, socioeconomic status is not a monolith. I am really grateful to continue to see this research on disaggregation because it is needed, whether we speak English or other languages. We are not all well educated with a certain income.

Also, does your research talk about the hate that is out there, hate crimes and the fears of reaching out for public connection, for those able to be out in the street as elderly Asian people. What other types of research do you all envision seeing?

***Dr. Mclemore:* Let me and Dr. Chen answer that question because you are absolutely right. There are no specific articles that are included that were about addressing directly the Stop Asian Hate and/or hate crimes that made it into the special edition. But there is one article in the regular issue of *Health Equity* because it came in after the submission deadline.**

**It is specific to the data set that is being collected at San Francisco State documenting hate crimes against Asian Pacific Islanders.**

***Dr. Chen:* Yes, and we do have one article in the special issue that deals with racism. There is an article by Dejan Su, who used the Health Ethnicity and Pandemic Survey and found out that 19% of non-Hispanic Asian and blacks reported being discriminated during the pandemic, but these levels were about 15% among Hispanics and only 3% among non-Hispanic whites.**

**I have to put a disclaimer that I am one of the coauthors.**

***Dr. Lett:* I am actually going to save your question for the end. During the last 5 min for everyone, let us discuss what do we need to see happen next? Because I really want to give Dr. Zhang the floor to go through her work.**

**Dr. Zhang:** I am Stacy Zhang. I am currently associate professor in the Division of Health Services Research at NYU Long Island School of Medicine. In October 2020, right before the election, we collaborated with the National Opinion Research Center at the University of Chicago, to conduct a nationally representative survey.

The survey is called the “Health Ethnicity and Pandemic (HEAP) Study.” It was jointly funded by the University of Nebraska Medical Center, Chinese Economists Society, and the Calvin J. Li Memorial Foundation. In that survey, we investigated a range of topics such as racial discrimination experiences, perceived racial bias during the pandemic, lifestyle changes, mental health status, health care utilization, and trust in media among 2709 adults aged ≥18 years.

The study sample was drawn from the representative AmeriSpeak Panel and we oversampled Asian Americans in our survey. So, we could do subgroup analyses by looking at South Asians, Southeast Asians, and East Asians separately.

In general, by racial and ethnic groups, we found Hispanic and Asian Americans had the highest prevalence of severe mental distress measured by the Kessler Psychological Distress Scale during the pandemic. But among Asian Americans, we found that Southeast Asians and South Asians had the highest prevalence of severe mental distress during the pandemic, which was higher than all other racial and ethnic subgroups.

This pattern suggested that it was really important to screen mental health status and other health-related conditions among Asian American subgroups because Asian Americans might be the most heterogeneous ethnic population. But we also acknowledged that there was a critical limitation in our survey study. We only used English and Spanish in this multiethnic survey. Unfortunately, we lacked the financial resources to use multiple Asian languages when we conducted the survey, and likely missed the opportunity to survey participants who spoke languages other than English and Spanish.

Our study had important public health implications. We should provide culturally competent services and interventions for Asian American subpopulations, by understanding their experiences, cultural backgrounds, coping mechanisms, and/or language barriers in accessing health care.

For instances, even before the pandemic, Asian Americans were less likely to use mental health services than non-Hispanic white population. Racial discrimination was associated with worse mental health status among Asian Americans. Cultural factors also play a role, as seeking mental health care was perceived as shameful and may be discriminated in their own communities.

Those issues occurred again during the pandemic and were exacerbated when they confronted new issues such as hate crimes, anti-Asian climate, and anti-immigrant comments on social media. Therefore, I think the first step for us to take action is that we need to disaggregate data on the AAPI community.

And the next step is, if we have enough financial support, we should use multiple Asian languages to reach out to the most vulnerable communities, and populations that we identified actually suffered most from mental health issues during the pandemic and beyond. I think we should work with health care systems, social workers, and leaders in those communities to develop culturally competent interventions on where to find essential resources, and how to cope with mental health issues.

**Dr. Yin:** I am Leena Yin. I am actually currently a family medicine resident at Swedish in Seattle, where I work in Seattle's Chinatown and care primarily for AAPI patients here in our Washington State community. And our article was a systematic review of the validity of the Chinese language PHQ-2 and PHQ-9.

The PHQ is one of the primary screening tools for major depression. It is a tool that most of us use a lot in our various practices. As somebody who practices a lot in Chinese, it occurred to me and to my study coauthor, do we take for granted that these tools are accurate when translated into different languages?

Do they have the same level of specificity and sensitivity in our patient populations that they would in English? I think it is a question that is important to ask and something, again, that we take for granted. Particularly for Chinese Americans, monolingual Chinese Americans living here in the United States, and for a lot of Asian American subgroups as well, we know that there is a lower rate of mental health need detection as well as access to services here in this country for a variety of reasons.

It is important to know that the diagnostic and screening tools that we are using are effective. That was the prompt for our study. We looked at different studies through September 2021 that evaluated the Chinese language PHQ-2, which is a brief screening tool, or PHQ-9, which is a more extensive screening tool and compared the results of those screenings with clinical interview diagnosis of depression.

We looked at over 500 articles and we pulled 20 that ultimately met our rigorous inclusion criteria. And we also pooled all of the data from those studies in addition to analyzing their quality and finally found that the pooled sensitivity and specificity of the PHQ-9 in the Chinese language were 88% and 87%, respectively, which were actually comparable with the rates when researchers use a PHQ-9 in English, because its original study sensitivity and specificity were 88%.

So, that is actually very good—it's comparable. And the PHQ-2 was similarly 84% and 87% pooled with a low risk of bias in all of the studies. However, something we use the PHQ-9 for is also grading depression. When somebody has a diagnosis of depression, how severe is it? And there were not many studies looking at the accuracy of the PHQ-9 as a depression grading tool in terms of severity, which is something that really informs our treatment regimen for these patients. And there is also some implication from the research that we looked at that the cutoff score for the PHQ-9 for whether somebody meets the cutoff or having some kind of depression versus not having depression may actually be different for our Chinese-speaking patients compared with our English-speaking patients.

And finally, we looked at 20 studies, but only 1 of them took place in the United States. And it was out of the Chinese community serving community health center in Boston. The remaining studies were abroad, mostly in mainland China, with some in Taiwan and Hong Kong. So, whether or not these data are truly applicable to folks who have lived in the United States for a long time remains to be seen. And obviously I would love to see this research done for other languages such as Vietnamese or Khmer. Obviously, every subgroup is different as I think is the recurring theme in this conversation.

***Dr. Lett:* Thank you, Dr. Yin. I think that validation speaks of this long-standing political aspect of science where we assume that the global minority white people are the default and do not take into account the importance of understanding in which ways people might diverge. But I also do not like to frame it as a divergence from white people; I would rather think of it tailored to specific communities. So, I appreciate you doing that. I think the standard for validation should be to develop instruments on diverse populations and then within those initial validation studies just to do some validations within all the different communities at the onset.**

***Dr. Mclemore:* One of the reasons why I love psychometric articles so much, and kudos on this work, is because one of the fundamental questions that I want to ask is, are the initial constructs that are being asked in these validated and reliable measures, are they even applicable?**

**Are these universal concepts, do they really hold true in the context of removing white supremacy? So for me, one of the big things that I loved about the articles that you selected in the methodology that you use, it was really more about not just trying to see are people trying to be resilient in using a mental health screening tool, but you really dug deeper to get at the actual questions that were being asked and the constructs that were being measured.**

**I think you are on point in terms of we need better tools that are not just responsive like, that is, in the context of white supremacy, are you experiencing depression or even defining depression as a pathological reaction to racism and white supremacy and that it is a resilient measure? You're spot on in terms of that. But I love the way the systematic review really turned the psychometric piece on its head to really make some determinations of really looking at not just the assets but the applicability of the underlying constructs that were being measured.**

**I think it also gives us a way forward, not just in the clinical space, but also in the translational space for those of us who are developing measures and what those shared metrics could look like.**

***Dr. Lett:* And the alternative to shared measures are measures that are not shared too, right? This is a discussion of intersectionality; I consider myself a burgeoning intersectionality theorist. There are some things that you cannot actually measure in white people that are relevant to the health of other people. You cannot measure the negative effects of white supremacy to white people. Actually, to be very specific, you cannot measure white supremacist discrimination. There is a lot of nuance in that discussion in white people, so having measures that are tailored to other communities is important. We do not always need to include white people if it is not something that they can be subject to in measurement.**

**Dr. Samoa:** My name is Ray Samoa. I am an endocrinologist in Southern California at the City of Hope. I am also the lead for the Data and Research Council for the National Association of Pacifica organizations. I have been so enamored with all the work that is being done on this issue because there are so many different facets regarding COVID-19 and vaccinations.

Our article looked at vaccine hesitancy in Native Hawaiian and Pacific Islanders. We were charged by congress with looking at the experience of communities of color. I was asked by a group from DePaul, the Asian American Psychology Association, to join them in doing a national survey.

Myself and Dr. Nia Aitaoto would oversee the Native Hawaiian and Pacific Islander survey. I think it was mentioned before how important it is to engage communities. So even though I am an academic researcher, my participation in this project was entirely as part of the community. We reached out to community-based organizations that have been doing work regionally for a long time with regard to chronic disease management.

They were the perfect engagers of the community to get feedback as to where they are, how do we reach them? What is the best mode? What kind of questions they would respond to and what was on their desk with regard to things that were concerning them? Based on their feedback, we were able to put forth our study design and really use the community to get respondents.

We were able to get about 1200 respondents who reflected the census of Pacific Islanders very well in the United States. We had good representation from the more populous Polynesian groups such as Samoans and Hawaiians and Tongans. We also had a fair number of respondents from our Marshallese, and we had a good response geographically.

So regionally, we know that many Pacific Islanders traditionally have been based in the west coast. But, recently since 2000 because of a heavy recruitment by agricultural industries, there has been a huge rise in Pacific Islanders in the south and the midwest. Our study showed respondents from those areas very well. What we found was there was a fair amount of vaccine hesitancy in Pacific Islanders.

But, as we mentioned before, Native Hawaiian Pacific Islanders are not a monolith. There were many differences among subgroups with regard to what drove their vaccine hesitancy. One of the things that we noticed has been published over and over again in the literature is that there are many sociodemographic descriptors that help drive a lot of people's thinking around COVID-19.

In some of the larger groups, we noticed that there is more hesitancy the lower one's educational attainment and income was. That reflected what was being reported nationally. But when you looked at subgroups more closely, geographically, we noticed that that did not hold true. There was still a fair amount of hesitancy regardless of income or educational attainment, which means that the filter that these subgroups are getting their information does not follow the same track lines as the general population, that they were getting their information from top to bottom through groups that may not have their best welfare at heart.

So, what we did was we translated that into work. The groups that the community-based organizations that looked for us for leadership in the academic area, in these areas of research, what they needed to do was to get resources to do something about these findings that if we were not getting—if how much you make or your educational attainment was not driving it, then there has to be something else.

We were able to leverage that information to engage these communities to go deeper into those that had a lot of high vaccine hesitancy in places such as Arkansas, in places such as Utah, in places where there is a red and blue divide. We were able to engage communities to set up vaccine clinics there, leveraging their trust equity to fight back against the messaging to get people vaccinated.

That is how we translated our research findings into action. Everything in the middle was all done through community. It was going back and telling them, “how do you want to do this study?” And then once we got the study results, “what do you want to do with these study results? What resources do you need to combat what we found?”

I think it made me a bigger fan of community-led research. I know we had a lot of talk about CPPR, community-based participatory research, but I will tell you as somebody that has been on both sides of the fence, CPPR still leverages toward the academic center. It still puts most of the bargaining chips on the side of the researcher. I think if we are going to get more salient research that has more effective impact on policy, we are going to need to let the community somehow be more involved in research development.

One of the things I think might help in that regard is having them pick their own champion, having them pick their own topics. I have talked about this at the NIH; having communities find their own champion makes researchers go and seek community out. It makes them develop relationships with community way before grants come out, way before topics are made popular because the leverage is finally on the community side.

***Dr. Lett:* One thing that I want to push on that you brought up a recurrent thing has been community. And I think you are getting to the point that community-based participatory research, the out-of-the-box version of it, tends to be extractive in the way that academia generally is. But I want to push people to think about participatory action research, which is a different paradigm that starts with the community and it is about action.**

**So, throughout the entire process it is not just extracting findings for research. It is actually a community-devised action that is the ultimate goal and your products and things are secondary. I think that is what you are actually talking about. That is something we should try to bring to the forefront. It is a community activist research framework in a way. And then another thing that I want to draw out is that with this community thread, this is perhaps—actually, not perhaps—this some of the most nuanced research I have ever seen about a racial ethnic minority and it cannot be lost that a lot of the people, all of the people who are authors, come from those communities.**

**This is a strong case for the inclusion of people, to borrow from disability justice, nothing about us without us. I think this is just a beautiful representation. There were no explicit conditions on who the identities were for the people who are in the special edition, but only people from those populations were able to get the rigor and the quality that we demanded at *Health Equity***.

**Dr. Montalvo:** I want to build on what you just stated. And I do think that because of the community participation with the researchers that we are sharing today, you also create pathways and you are probably one of the first role models for people who may work in these communities to view themselves at this next level of their development because I know that was the case for me. And I think you are spot on.

As someone who works at the National Association of Community Health Centers, we get inundated all the time with requests. Oftentimes, the questions are already formulated, and we just turn them away because they are not really connected to the communities we serve.

***Dr. Lett:* Patricia Hill Collins and her work intersectionality as critical theory says that everyone is a theorist. Everyone around us is a scientist. We are all building heuristics on how the world moves and how things happen to us and to people around us. Community-based people have just as much knowledge and just as much science to contribute as the rest of us.**

**I think the dichotomy between community and academia is false and only the result of sequestration of resources under a white supremacist capitalist framework.**

***Dr. Chen:* I want to continue with the discussion on community engagement. I will just give one example. In the NIH All of Us program, there are only about 3% to 4% of AANHPIs despite more than 7% of the general population being AANHPI.**

**So, that is a significant under-participation in this research group. And I think by engaging the communities by their contribution working from the AANHPI research community to engage them, I think there are more opportunities for a more representative panel over all of us. And in that line, there will be more research findings that can help this community.**

**Engaging the community is key. That is one of the messages Dr. McLemore, Dr. Ma, Dr. Zhang, and I tried to convey in the editorial summary of the special issue.**

**Dr. Samoa:** I love what you said about community action research. Full disclosure, I am an endocrinologist. I am a diabetes specialist. I really had no business going into the COVID-19 space, but the community said, you are a representative in this space.

***Dr. Mclemore:* I just want to say and reiterate as the editor-in-chief of *Health Equity* that this is not the last special edition on AAPI health. Let us be clear. Let us not act like we are not going to do another one, because we are. And I want to thank you Dr. Lett for your leadership and your skills in hosting this first of what will be many Twitter spaces with our broader participants and community.**

**I am grateful for everyone who listened, who joined. The recording will be pinned to the *Health Equity* Journal Twitter feed.**

